# Exercise Training, Lymphocyte Subsets and Their Cytokines Production: Experience of an Italian Professional Football Team and Their Impact on Allergy

**DOI:** 10.1155/2014/429248

**Published:** 2014-06-23

**Authors:** Stefano R. Del Giacco, Marco Scorcu, Federico Argiolas, Davide Firinu, G. Sergio Del Giacco

**Affiliations:** ^1^Department of Medical Sciences “M. Aresu”, University of Cagliari, Asse Didattico “E1”, Cittadella Universitaria, Monserrato, 09042 Cagliari, Italy; ^2^National Health Service, ASL 6, 09025 Sanluri, Italy; ^3^Cagliari Calcio Football Club, 09123 Cagliari, Italy; ^4^National Health Service, ASL 8 Public Health Services, “Businco” Hospital, 09121 Cagliari, Italy

## Abstract

*Background*. In recent years, numerous articles have attempted to shed light on our understanding of the pathophysiological mechanisms of exercise-induced immunologic changes and their impact on allergy and asthma. It is known that lymphocyte subclasses, cytokines, and chemokines show modifications after exercise, but outcomes can be affected by the type of exercise as well as by its intensity and duration. Interesting data have been presented in many recent studies on mouse models, but few studies on humans have been performed to check the long-term effects of exercise over a whole championship season. *Methods*. This study evaluated lymphocyte subsets and their intracellular IL-2, IL-4, TNF-*α*, and IFN-*γ* production in professional football (soccer) players, at three stages of the season, to evaluate if alterations occur, particularly in relation to their allergic status. *Results and Conclusion*. Despite significant mid-season alterations, no significant lymphocyte subclasses count modifications, except for NKs that were significantly higher, were observed at the end. IL-2 and IL-4 producing cells showed a significant decrease (*P* = 0.018 and *P* = 0.001, but in a steady fashion for IL-4), confirming the murine data about the potential beneficial effects of aerobic exercise for allergic asthma.

## 1. Introduction

In recent years, the search for new training methods to obtain maximum performance in sports has become of fundamental interest to trainers and athletes, and the study of exercise immunology has become part of this strategy. In fact, exercise-induced immune modifications may show a wide range of changes [1, 2], influencing positively or negatively an athlete's health. On the one hand, they may lead to an increased risk of infections (the “open window” theory) [3]; on the other hand, the positive effect of regular exercise on immune changes has been recently demonstrated in findings on experimental mouse models [4]. Both innate and acquired immunity are influenced by exercise. Studies performed to date in the field of “exercise immunology” explored many variables such as “acute” versus “prolonged” exercise, different sports, age, and recreational versus professional athletes. The effect of chronic exercise has been studied longitudinally after at most few months of training and often in subjects previously untrained or affected by chronic diseases [5, 6].

During acute exercise, modifications in mononucleated blood cells occur [7]. Increased levels of all the lymphocyte subsets have been found and some authors have reported a fall in the CD4/CD8 ratio [6]. At the end of the exercise, values fall to below-normal levels; according to a number of authors both the duration and the reduction depend on the intensity and duration of the exercise itself [1]. Natural Killer (NK) cells, the most responsive immune cells to acute exercise, show a striking exercise-induced increase, whereas their cell count drops to below half the normal level after exercise [8, 9]. Their cytotoxicity increases during exercise, but it is depressed after exercise, probably augmenting the susceptibility to infections [10–12].

The production of cytokines increases in response to acute exercise. Proinflammatory cytokines (IL-1, IL-6 and TNF-*α*) concentrations rise after prolonged exercise (a 20 Km run, for instance); increased IL-1*β* is found in the muscular tissue; and increased IL-1 activity can be noted (muscular proteolysis?), even if a suppression of their production one hour after exercise has been reported by other authors [6, 13]. Recent cross-sectional data described increased NGF serum levels in top athletes [14]. Additionally, recent findings are leading to interesting new theories on the anti-inflammatory effect of regular, moderate exercise. Lower levels of Th-2 cytokines and enhanced Th-1 and T-reg responses in murine models of asthma performing aerobic exercise versus sedentary mice have been reported in recent studies [4, 15, 16].

Limited data have been published on the immunological effects of regular exercise ex vivo in top athletes and in* amateurs* during a longer time span, such as an entire training season, avoiding overtrained subjects [17, 18]. Therefore, the aim of this study was to evaluate lymphocyte subsets and their intracellular IL-2, IL-4, TNF-*α*, and IFN-*γ* production in professional soccer players during an entire league championship. We obtained blood samples at three stages of the championship season, to verify whether alterations occur, and what the clinical relevance of these modifications could be, in particular in relation to their allergic status.

## 2. Materials and Methods

All players (29 athletes) of an Italian “Serie A” (Premier League) football (soccer) team underwent blood sampling (at rest, before training, and 72 hours after a match) before the start of season (June), halfway through the season (January) and at the end of season (May), respectively. All the subjects were checked through the* AQUA* ^*©*^ validated allergy questionnaire for athletes [19]. Informed consent was obtained from all participants and the study protocol was approved by the Ethics Committee of the University Hospital. Analysis of the collected samples was performed using a Becton Dickinson FACSCalibur Immunocytometry System (Franklin Lakes, USA). For lymphocyte subsets, a Multi-Test IMK Kit (Becton Dickinson), that is a four-colour direct immunofluorescence reagent kit for identifying and determining the percentages and absolute counts of mature human lymphocyte subsets, was used. Fluorochrome-labelled antibodies (CD45RA-FITC, CD45R0-FITC, CD62L-PE, CD3PerCP, CD4-APC, and CD8-APC) that bind specifically to the leukocyte surface antigens were employed: T Lymphocytes (CD3+), B lymphocytes (CD19+), helper/inducer T lymphocytes (CD3+, CD4+), suppressor/cytotoxic T lymphocytes (CD3+, CD8+), and Natural Killer lymphocytes (CD3- CD16+ and NK or CD56+). CD4+ T-cells were studied for intracellular cytokines production allowing us to distinguish the functionally polarized cells through their cytokine secretion pattern.

For each of the athletes, data regarding the proportion of cytokines producing lymphocytes (IL-2, IFN-*γ*, TNF-*α*, and IL-4) and the lymphocyte subpopulations (absolute count and proportion of NK, CD3, CD4, CD8, and CD19) were compared by analysing their variation in the three consecutive blood samples.

## 3. Statistical Analysis

Considering the number of cases (<30), in order to test the significance of the differences, the Wilcoxon Signed Ranks Test was used. Values of two consecutive samples were compared (1st sample versus 2nd sample, and 2nd sample versus 3rd sample); furthermore, values obtained at the beginning of the season were compared with values obtained at the end (1st sample versus 3rd sample). *P* < 0.05 was considered as significant. Percentages, medians, interquartile ranges, and “*P* values” are shown in the results tables. Data were processed through SPSS (Release 20.0, Copyright ©SPSS Inc., Chicago, IL, USA) for Windows.

## 4. Results

All the subjects were males, aged 17–30 years (mean age; 24.3 yrs). Of the 29 athletes that at any moment could have been part of the team, 16 underwent 3 blood samplings, 7 were present at least 2 consecutive samplings, 2 performed two nonconsecutive samplings (the first and the last), and 4 underwent only one blood sampling. Data of the subjects from the 3 first groups (25 athletes, 86% of the whole population studied) were taken into consideration to perform the statistical analysis. The last group (4 athletes, 14%) was not considered for statistical purposes. Out of the whole group, 8 (28%) had respiratory allergies (AQUA questionnaire, further confirmed by history, symptoms, and skin prick tests) and were undergoing treatment with inhaled corticosteroids and Beta-2 agonists. Seven out of the eight allergic athletes were included in the 25 subjects providing samples for the final analysis (Table 1).

**Figure 1:**
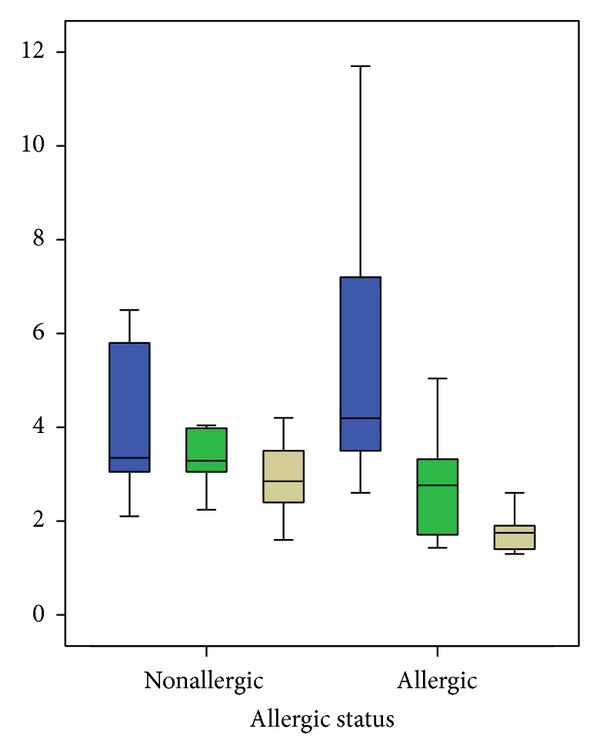
IL-4 producing lymphocytes % (nonallergic versus allergic athletes) during the three timepoints of the season. The boxplots highlight a constant decrease of IL-4 producing lymphocytes from the beginning till the end of the championship. In the allergic group the final IL-4 producing cells proportion is lower even with higher basal values. Exact values can be checked in Table 3.

The proportion of IL-2 and IL-4 producing lymphocytes showed statistically significant changes in each comparison between the three different moments of the championship (see Table 2). A significant decrease in IL-2 producing cells was seen at the middle of the championship. At the end of the observation period, cell percentage was still statistically significantly lower despite a slight increase (Table 2). A different pattern was observed with the IL-4 producing cells: a continuous and significant decrease from the beginning of the study to the end of the championship was witnessed (Table 2). The allergic athletes were the most involved in the IL-4 producing cell decrease (Figure 1). A comparison between allergic and nonallergic subjects is reported in Table 3. The only significance obtained for TNF-*α* and IFN-*γ* producing cells is related to their increase between the middle of the championship and the last determination (Table 2).

Comparisons between the cells absolute counts (Table 4) showed a similar pattern. All the lymphocyte subclasses counts at the middle of the championship were much higher than at the beginning (*P* always < 0.005). At the end of season, all the absolute counts showed a significant drop in comparison to the middle of the season (*P* always < 0.05), being much similar to those noticed at the beginning of the championship. CD3, CD4, and CD8 absolute counts did not show significant changes comparing the 1st to the 3rd samples (*P* > 0.05). NK cells absolute count remained significantly higher at the end of the championship (*P* = 0.002). Regarding the differences between the percentages of lymphocytes subclasses, a statistically significant increase in the NK% between the beginning and the middle of the championship is noticeable (*P* < 0.001); this increase remains significant at the end of the championship (*P* = 0.030). Contrarily, a significant decrease in the CD3% (*P* = 0.001) and a slight, but significant, decrease in the CD4% (*P* = 0.024) is recorded (1st versus 2nd sample); this decrease remains significant at the end of the championship (1st versus 3rd sample, CD3% *P* = 0.033 and CD4% *P* = 0.049). No significant changes were recorded for CD8%.

## 5. Discussion

The modifications highlighted in this study suggest that athletes performing a regular training program during the entire season, such as that performed by professional athletes, do not have a reduction of cell absolute counts. NK absolute count and percentage increase significantly. To our knowledge, data obtained in such a long timescale (one year) are scarce; the results reported in the present study may challenge, at least in part, the concept that the NK decrease in the recovery phase (a few hours after competitions) may be linked to an increased susceptibility to viral infections [20]. In particular, the reported NK reduction is clearly a transient phenomenon as it does not persist at a larger scale of observation such as the one presented in our data at six months and one year (Table 4). This finding could be in agreement with data reported in the literature showing an increased NK cell activity and no changes in upper respiratory infections incidence in professional athletes versus nonathletic controls [18, 21].

A reduced production of IL-2 in these athletes was observed: it could be speculated that the cells might become less reactive to antigenic stimuli [22] with a relative decreased immune reactivity. IL-2 is essential for extrathymic homeostasis of Treg Lymphocytes, which are central regulators also of allergic inflammation. However, a treatment of allergen-sensitized mice with recombinant IL-2 paradoxically increased airway eosinophilia and tissue inflammation in one study [23]. Therefore, our finding of reduced IL-2 production should be further investigated also for its effects on the Treg lymphocytes compartment.

Furthermore, in our study IL-4 producing cells are significantly reduced at the end of the season, in particular as regards the allergic athletes (Figure 1 and Table 3).

It is known that, among the several cytokines involved in the allergic asthma pathogenesis, IL-4 plays a fundamental role [24, 25]. Allergic individuals show a “TH2 family” cytokine pattern. The elaboration of TH-2 effector cytokines is dependent on IL-4 and IL-25 and their respective transcription factors GATA-3 [26], STAT-6 [27], and c-maf [28], whereas negative transcription occurs through cytokines that favour TH-1, such as IL-18 [29].

The growing number of studies on the murine model of allergic asthma shows that low to moderate intensity aerobic exercise decreases eosinophilic and lymphocytic inflammation in mice exercising for 4 weeks, 5 days a week, at 50% exercise capacity [4]. Aerobic exercise also seems to reduce airway remodeling, with reduced airway smooth muscle hypertrophy and hyperplasia [4, 30] (even if effects on remodeling were a matter of debate in another animal study [31]), a reduction in leukocyte infiltration, proinflammatory cytokine production, adhesion molecules expression [32], and enhanced regulatory T-cell (Treg) responses [16]. Aerobic exercise also shows an anti-inflammatory effect in mice exposed to air pollution [33]. A single session of moderate aerobic exercise can decrease airway inflammation (but not responsiveness) in mice, with a downregulation of inflammatory mediators' genes expression and Th-2 derived cytokines production [15]. Similar findings have also been demonstrated in one study on humans, where a reduction in neutrophils count in patients with chronic inflammatory conditions was observed [34]. Furthermore, a recent systematic review concludes that physical training improves airway inflammation in animal asthma models [35] and another study shows that it does not increase airway inflammation in children [36].

Our data, demonstrating for the first time a reduction of the IL-4 producing cells in professional athletes over a long time span of observation, could be in agreement with the data coming from the murine studies previously cited.

## 6. Conclusion

This is probably the first study to demonstrate the immunological modifications that occur in a long-term, real-life setting in professional athletes, and this undoubtedly represents one of its strengths. However, there are some limitations in this study, linked to the peculiar characteristics of professional athletes. The narrow age range and the male-only population can reduce the interpersonal variability. Furthermore, the lack of a sedentary control population may also reduce the general value of these results, even if, to our knowledge, no significant physiologic seasonal changes in the parameters studied have been described in the general population [37–39].

In summary, the findings in this study suggest that normal lymphocyte percentages and counts are not impaired by a regular training program, such as that performed by professional trained athletes. The decrease of IL-4 producing cells can be considered interesting as one of the factors for the improvement of symptoms in allergic and asthmatic people performing a regular training program [40]. Aerobic exercise training seems to be beneficial for allergic inflammation, and it may be suggested as a comprehensive part of the prevention and therapy strategies for asthmatics.

## Figures and Tables

**Table 1:** Sensitizations in the allergic athletes selected (skin prick test).

Sample number	Age	Sensitization(s)	Symptoms
15	25	Pellitory, Ragweed, *Cladosporium, *Ash tree	Rhinoconjunctivitis
16	26	House dust mites, Dog	Rhinitis
21	20	Grass, Pellitory, Cat	Asthma + Rhinitis
27	24	Grass, Olive, *Chenopodium *	Asthma + Rhinitis
29	26	House dust mites, Mugwort, *Alternaria *	Asthma + Rhino-conjunctivitis
31	27	Grass, Olive	Rhinoconjunctivitis
33	29	House dust mites, Grass, Cypress	Asthma + Rhinoconjunctivitis

**Table 2:** Cytokines producing lymphocytes (percentage)*.

	1st sample	*P* ^§^ (1st-2nd)	2nd sample	*P* ^§^ (2nd-3rd)	3rd sample	*P* ^§^ (1st–3rd)
IL-2	22.80 (14.80–26.65)	***0.001***	11.63 (9.57–15.39)	***0.001***	16.20 (12.20–20.25)	***0.018***
TNF-*α*	31.00 (24.05–39.05)	*0.107 *	25.88 (20.01–31.08)	***0.015***	29.75 (22.65–33.75)	*0.184 *
IFN-*γ*	12.30 (10.10–15.70)	*0.445 *	12.30 (9.33–15.23)	***0.008***	14.70 (10.90–18.35)	*0.569 *
IL-4	4.19 (3.03–5.70)	***0.014***	3.23 (2.21–3.51)	***0.001***	2.45 (1.68–2.98)	***0.001***

*Data are expressed in medians (interquartile range).

^§^Wilcoxon Signed Ranks Test.

**Table 3:** Cytokines producing lymphocytes, allergic and nonallergic subjects (percentage)*.

	1st sample	*P* ^§^ (1st-2nd)	2nd sample	*P* ^§^ (2nd-3rd)	3rd sample	*P* ^§^ (1st–3rd)
Allergic (7 subjects)						
IL-2	20.80 (10.80–26.20)	***0.063***	12.16 (9.87–16.06)	***0.028***	17.25 (13.50–24.95)	*0.463 *
TNF-*α*	28.90 (16.50–36.60)	*0.612 *	25.88 (20.01–33.11)	*0.345 *	31.90 (24.38–35.10)	*0.753 *
IFN-*γ*	14.40 (12.30–15.40)	*0.091 *	12 (9.33–14.68)	***0.028***	15.30 (11.18–19.98)	*0.500 *
IL-4	4.19 (2.60–7.20)	***0.042***	2.21 (1.58–3.32)	***0.028***	1.75 (1.38–2.08)	***0.028***

Nonallergic (18 subjects)						
IL-2	22.95 (15.30–27.75)	***0.003***	10.98 (9.35–15.06)	***0.006***	16.20 (12.20–20.00)	***0.019***
TNF-*α*	31.55 (26.98–41.38)	***0.008***	26.36 (18.55–31.06)	***0.022***	28.45 (22.55–32.18)	***0.041***
IFN-*γ*	11.55 (9.60–16.83)	*0.754 *	13.06 (9.34–15.73)	*0.109 *	13.70 (10.30–16.90)	*0.423 *
IL-4	3.85 (3.04–5.65)	*0.091 *	3.29 (2.82–3.82)	***0.009***	2.70 (2.03–3.43)	***0.021***

*Data are expressed in medians (interquartile range).

^§^Wilcoxon Signed Ranks Test.

**Table 4:** Peripheral lymphocyte subclasses (percentage and absolute count)*.

	1st sample	*P* ^§^ (1st-2nd)	2nd sample	*P* ^§^ (2nd-3rd)	3rd sample	*P* ^§^ (1st–3rd)
Percentage						
NK	13 (8–16)	***<0.001***	19 (14–22)	*0.176 *	18 (12–24)	***0.030***
CD3	72 (67–78)	***0.001***	67 (64–72)	*0.058 *	67 (62–72)	***0.033***
CD4	42 (37–49)	***0.024***	41 (37–44)	*0.639 *	40 (38–44)	***0.049***
CD8	26 (21–30)	*0.916 *	24 (20–26)	*0.849 *	24 (20–26)	*0.223 *

Absolute count						
NK	232 (196–288)	***<0.001***	519 (423–662)	***<0.001***	375 (247–510)	***0.002***
CD3	1416 (1267–1678)	***0.001***	1862 (1682–2369)	***0.002***	1442 (1294–1707)	*0.679 *
CD4	876 (717–990)	***0.002***	1135 (916–1245)	***0.010***	868 (749–1060)	*0.943 *
CD8	497 (419–571)	***<0.001***	703 (518–865)	***0.004***	521 (468–568)	*0.071 *

*Data are expressed in medians (interquartile range).

^§^Wilcoxon Signed Ranks Test.
